# Pricing appraisal of anti-cancer drugs in the South East Asian, Western Pacific and East Mediterranean Region

**DOI:** 10.1186/s12885-017-3888-y

**Published:** 2017-12-28

**Authors:** Shahrzad Salmasi, Kah Seng Lee, Long Chiau Ming, Chin Fen Neoh, Mahmoud E. Elrggal, Zaheer-Ud- Din Babar, Tahir Mehmood Khan, Muhammad Abdul Hadi

**Affiliations:** 1 Collaboration for Outcomes Research and Evaluation, Faculty of Pharmaceutical Sciences, University of British Columbia (UBC), Vancouver, Canada; 2 Pharmaceutical Services Division, Ministry of Health, Petaling Jaya, Selangor Malaysia; 3 Unit for Medication Outcomes Research and Education (UMORE), School of Medicine, University of Tasmania, Hobart, Australia; 4 Faculty of Pharmacy, Universiti Teknologi MARA, Puncak Alam, Selangor Malaysia; 5 College of Pharmacy, Umm-Al-Qura University, Makkah, Saudi Arabia; 6 Department of Pharmacy, School of Applied Sciences, University of Huddersfield, Huddersfield, UK; 7 School of Pharmacy, Monash University Malaysia, Subang Jaya, Malaysia; 8 Leicester School of Pharmacy, De Montfort University, Leicester, UK

**Keywords:** Anti-cancer drugs, Pricing, South-East Asia, Western Pacific, Eastern Mediterranean

## Abstract

**Background:**

Globally, cancer is one of the leading causes of mortality. High treatment cost, partly owing to higher prices of anti-cancer drugs, presents a significant burden on patients and healthcare systems. The aim of the present study was to survey and compare retail prices of anti-cancer drugs between high, middle and low income countries in the South-East Asia, Western Pacific and Eastern Mediterranean regions.

**Methods:**

Cross-sectional survey design was used for the present study. Pricing data from ten counties including one from South-East Asia, two from Western Pacific and seven from Eastern Mediterranean regions were used in this study. Purchasing power parity (PPP)-adjusted mean unit prices for 26 anti-cancer drug presentations (similar pharmaceutical form, strength, and pack size) were used to compare prices of anti-cancer drugs across three regions. A structured form was used to extract relevant data. Data were entered and analysed using Microsoft Excel®.

**Results:**

Overall, Taiwan had the lowest mean unit prices while Oman had the highest prices. Six (23.1%) and nine (34.6%) drug presentations had a mean unit price below US$100 and between US$100 and US$500 respectively. Eight drug presentations (30.7%) had a mean unit price of more than US$1000 including cabazitaxel with a mean unit price of $17,304.9/vial. There was a direct relationship between income category of the countries and their mean unit price; low-income countries had lower mean unit prices. The average PPP-adjusted unit prices for countries based on their income level were as follows: low middle-income countries (LMICs): US$814.07; high middle income countries (HMICs): US$1150.63; and high income countries (HICs): US$1148.19.

**Conclusions:**

There is a great variation in pricing of anticancer drugs in selected countires and within their respective regions. These findings will allow policy makers to compare prices of anti-cancer agents with neighbouring countries and develop policies to ensure accessibility and affordability of anti-cancer drugs.

**Electronic supplementary material:**

The online version of this article (10.1186/s12885-017-3888-y) contains supplementary material, which is available to authorized users.

## Background

Earlier diagnosis and longer treatment durations contribute to rising expenditure on medicine for cancer care. Access to cancer treatment can be a challenge, since it is significantly affected by cost, particularly in low and middle-income countries. According to the Global Oncology Trend Report [1], global spending on cancer medications rose from $75 billion in 2010 to $100 billion in 2014, 10.3% rise in spending. Medication cost is a strong predictor of adherence [2, 3] with the risk of cost-related non-adherence being higher for those with lower income and higher out-of-pocket (OOP) drug spending [3].These growing costs inevitably provoke concern regarding the financial burden experienced by cancer patients [1]. This concern is even more prominent in Asia because it is home to half of the world’s extremely poor population [4].

Asia accounts for 60% of the world population and 50% of the global burden of cancer [4]. The projected increase in cancer incidence is predicted to be most significant in low and medium—income countries in Asia [4, 5].

Asia is very heterogeneous in terms of healthcare systems. With the exception of a few high-income countries such as Israel, Kuwait, Qatar, the Republic of Korea, Singapore, and the United Arab Emirates who enjoy well-developed health services, the vast majority of the Asian people face a substantial cancer burden because cancer care remains a low priority in healthcare planning and expenditure [4]. In these countries, over 60% of the total healthcare expenditure comes from private resources, of which more than 80% is direct out of pocket payments, with catastrophic results for most families in these countries [4]. Similarly, in the Middle East, spending per capita on cancer drugs is considerably less than in Europe or the US [6]. The cancer drug expenditure as a percentage of total drug expenditure is very low in Middle Eastern countries [6].

In this study, we aim to build on the existing body of work by providing comparable cancer drug retail prices across countries in the South-East Asian [SEA], Western Pacific (WP) and Eastern Mediterranean (EM) regions. A review based approach utilising selective content analysis has been adopted to achieve the objective of this paper. A previous study on comparing cancer drug prices focused on 18 high-income countries, member of economic co-operation and development (OECD) countries, in Europe and Oceania [7]. To our knowledge, this is one of the very first initiatives taken to compare the retail price of cancer drugs across countries in the South-East Asia, Western Pacific and Eastern Mediterranean regions. By analysing and comparing the unit prices across Asian countries with differing gross national income (GNI) per capita, we are hoping to assist in improving procurements, price negotiations, and location of new supply sources, and ultimately to create an opportunity for patients in Asia to gain access to more affordable cancer treatments.

## Methods

### Country selection

The following criteria were adopted for the inclusion of countries:

- Geographically located in the South-East Asian, Western Pacific or Eastern Mediterranean regions
- Availability of drug pricing data published by respective pricing/health authorities.

Based on the inclusion criteria, Thailand was the only eligible country from South-East Asia, Malaysia and Taiwan were included from the Western Pacific, and Oman, Pakistan, the United Arab Emirates, Lebanon, Egypt, Saudi Arabia and Bahrain from the Eastern Mediterranean region. Countries in the Asia Pacific [Australia and New Zealand] were excluded from this study, despite being part of the Western Pacific region, as they had already been covered in previous studies [7]. The included countries were classified into Low income [LIC], low middle income [LMIC], high middle income [HMIC] and high income [HIC] countries based on their GNI per capita, using the cut off points provided by the world bank website [8]. The United Nation’s 3-letter standard abbreviations [ISO ALPHA-3 code] were used to represent country names in Tables 1, 2 and 3. [9]

**Table 1:** Data sources used in this study

Countries	Data sources	Specification
UAE	United Arab Emirates Ministry of Health Drug Department URL:http://www.cpd-pharma.ae/downloads/Price-List-February/MoH-Price-List-as-on-07-Feb-2015.pdf	The data was retrieved from MOH, drug department. The Document includes both imported and generic drugs and it was last updated on Feb 2015.
Bahrain	National Health Regulatory Authority URL: http://www.nhra.bh/SitePages/View.aspx?PageId=42	The Drug Price List includes both innovator and generic medicines prices. Last updated on 14 March 2016
Taiwan	National Health Insurance Administration, Ministry of Health and Welfare URL: http://www.nhi.gov.tw/query/Query1.aspx	The data was retrieved from Ministry of Health and Welfare of Taiwan. The Document includes both imported and generic drugs and it was last updated on June 2016.
Thailand	Drug And Medical Supply Information Center, Ministry of Public Health URL: http://dmsic.moph.go.th/dmsic/index.php?p=1&type=3&s=3&id=p_drug_normal_en&lang=en	The Drug Reference Price list provides the medical supplies prices for commercial sector. Last updated on March 2015.
Malaysia	Pharmaceutical Services Division, Ministry of Health URL: http://www.pharmacy.gov.my/v2/en/apps/drug-price	The Consumer Price Guide provides retail price list to serve as guidance to patients when purchasing medicines.
Lebanon	Ministry of Public Health URL: http://www.moph.gov.lb/Drugs/Pages/Drugs.aspx	The data was retrieved from the MoPH Drugs Public Price List. Last updated on 16 February 2016
Oman	Ministry of Health https://www.moh.gov.om/en/web/dgpadc/resources	The data was retrieved from the Ministry of Health, Sultanate of Oman website from the list of registered drugs. Last updated on 17–05-2016.
Pakistan	PharmaGuide book, Pakistan edition, 24th edition.	The pricing data was retrieved PharmaGuide Book. This handbook is published annually providing essential prescribing and trade information. The pricing data was retrieved from the latest edition (24th) published on March 2016.

**Table 2:** Background information about drugs included in the analysis^a^

Generic drug name	Product name	FDA approved indications	Selected presentation	Country coverage		Unit price is price of:
				#	Missing data	
Abiraterone acetate	Zytiga	Metastatic castration-resistant prostate cancer	120 tablets 250 mg	7	PAK, MYS, LBN	1 Tab
Bevacizumab	Avastin	NSCLC Metastatic Colorectal cancer, Glioblastoma Metastatic Renal cell carcinoma Metastatic Her2 negative breast cancer Metastatic cervical cancer.	One 4 ml vial containing 25 mg/mL concentrate for solution for infusion.	6	MYS, SAU, OMN, LBN	4 ml vial
Bortemozib	Velcade		One vial containing 3·5 mg powder for solution for injection.	7	EGY, PAK, SAU	1 vial
Cabazitaxel	JEVTANA®	Metastatic hormone refractory prostate cancer	One vial containing 60 mg concentrate and solvent for solution for infusion.	4	EGY, PAK,THA, SAU, BHR,TWN,	1 vial
Cetuximab	Erbitux	*K-ras* wild-type, *EGFR*-expressing metastatic colorectal cancer. Recurrent/metastatic head and neck cancer	One vial containing 5 mg/mL solution for infusion.	4	ARE, EGY, PAK, SAU,OMN, BHR	1 vial
Denosumab	Prolia	Unresectable giant cell tumor of bone in adults and skeletally mature adolescents	One pre-filled syringe containing 60 mg solution for injection	6	PAK, MYS, SAU, BHR	One pre-filled syringe
Erlotinib HCl	Tarceva	Non-small cell lung cancer. Pancreatic cancer.	30 film-coated tablets 150 mg	7	MYS, SAU, LBN	1 film-coated Tab
Everolimus	Afinitor	Subependymal giant cell astrocytoma. HER2-negative breast cancer. Progressive neuroendocrine Tumors of Pancreatic origin. Advanced renal cell carcinoma.	30 tablets 10 mg	8	PAK, MYS,	1 Tab
Gefitinib	Iressa	Non-small cell lung cancer	30 film-coated tablets 250 mg	7	EGY, SAU,PAK	1 film coated Tab
Gemcitabine	Gemita (Atco)	Ovarian cancer Breast cancer. NSLCLC Pancreatic cancer	1 vial containing 1 g powder for solution for infusion.	9	SAU	1 vial
Imatinib Mesylate	Glivec (Novertis)	Dermatofibrosarcoma protuberans. Gastrointestinal stromal tumor. Myelodysplastic/myeloproliferative neoplasms. Systemic mastocytosis. Chronic Eosinophilic leukemia. Chronic Myelogenous Leukemia	60 film-coated tablets 100 mg	9	BHR	1 film coated Tab
Interferon Alpha - 2B	Interon A (Schering Plough	AIDS related Kaposi Sarcoma. Hairy cell leukemia. Melanoma. NHL	One multi-dose pen containing 3 million IU/0·5 mL solution for injection.	6	THA, EGY, MYS,OMN	One multi-dose pen
Lapatinib ditosylate	Tykerb FC	HER2 positive breast cancer,	70 film-coated tablets 250 mg	7	MYS, BHR, TWN	1 film coated Tab
Nilotinib	Tasigna	CML	112 capsules 150 mg	8	PAK, MYS	1 Cap
			112 capsules 200 mg	6	THA,LBN, OMN, BHR	1 Cap
Paclitaxel Albumin	Intaxel 30 mg/5 ml inj	NSCLC Breast cancer pancreatic cancer	One vial containing 5 mg/ml powder for suspension for infusion.	6	ARE, EGY, SAU, BHR	1 Vial
Panitumumab	Vectibix 400 mg/20 ml IV	Colorectal Cancer	One vial containing 20 mg/ml concentrate for solution for infusion	6	THA, PAK, MYS, TWN	1 Vial
Pazopanib	votrient		30 film-coated tablets 200 mg	8	PAK, MYS	1 film coated Tab
Pemetrexed Disodium Heptahydrate	Alimta (Eli Lilly)	non-squamous NSCLC Malignant pleural mesothelioma	One vial containing 20 mg/ml solution for injection.	9	OMN	1 Vial
Sorafenib	Nexavar (Bayer Schering)	Liver cancer Kidney cancer Thyroid cancer	60 film-coated tablets 200 mg.	5	THA, MYS, OMN, BHR,LBN	1 film coated Tab
Sunitinib malate	SUTENT (Pfizer)	Kidney cancer Gastointestinal stromal tumour Pancreatic Neuroendocrine tumours	28 capsules 12.5 mg	7	OMN, LBN, BHR	1 Cap
			28 capsules 25 mg	4	THA, LBN, MYS, OMN, BHR, TWN,	1 Cap
			28 capsules 50 mg	6	THA, LBN, MYS, OMN,	1 Cap
Trastuzumab	Herceptin (Roche)	Her2 over expressing breast cancer Her2 over expressive Gastric or Gastroesophageal junction Adenocarcinoma	One vial containing 440 mg powder for concentrate for solution for infusion	4	THA, LBN, MYS, SAU,OMN, BHR	1 Vial
			One vial containing 150 mg powder for concentrate for solution for infusion	4	ARE, EGYP, PAK, LBN, MYS, TWN,	1 Vial
Zolendronic acid	ZOLDIC	Multiple Myeloma	One vial containing 4 mg/5 ml concentrate for solution for infusion	4	ARE, LBN, MYS, OMN, BHR, TWN	1 Vial

^a^The National Cancer Institute. A to Z List of Cancer Drugs. USA: The National Institutes of Health U.S. Department of Health and Human Services, 2015

**Table 3:** PPP-adjusted prices of the selected cancer originator drugs in the 10 surveyed countries converted to USD

		Low middle income		High middle income			High income						
Generic name	Presentation	PAK($)	EGY($)	MYS($)	LBN($)	THA($)	TWN ($)	OMN ($)	BHR ($)	ARE($)	SAU($)	Mean unit price for drugs	High/Low ratio
Abiraterone acetate 250 mg	120 tab	NA	9724·22	NA	NA	NA	NA	NA	NA	8470·08	8867·61	70·17	1·73
	1 tab	NA	81·04	NA	NA	52·02	59·10	90·21	64·33	70·58	73·90		
Bevacizumab 25 mg/ml	Inj. 100 mg (4 ml)	NA	1233·18	NA	NA	1473·00	602·10	4122·80	767·00	1157·95	NA	1559·50	6·85
Bortemozib3.5 mg	1 vial	NA	NA	3622·22	2243·96	4630·12	2443·02	3878·90	2771·38	2560·04	NA	3164·22	2·06
Cabazitaxel 60 mg	1 vial	NA	NA	11,492·5	7112·50	NA	NA	17,304·95	NA	11,421·76	NA	11,832·93	2·43
Cetuximab 5 mg/ml	1 vial	NA	NA	1100·49	1850·55	997·12	456·25	NA	NA	NA	NA	1101·10	4·06
Denosumab 60 mg/ml	1 ml prefilled syringe	NA	798·21	NA	607·36	934·69	417·41	757·00	NA	665·27	NA	696·66	2·24
ErlotinibHCl 150 mg	30 film coated tab	7315.34	11,659·19	NA	NA	NA	NA	NA	NA	6837·45	NA	194·84	4·93
	1 film coated tab	243.84	388·64	NA	NA	160.54	78.76	152.5789	111.62	227.915	NA		
Everolimus 10 mg	30 tab	NA	8565·02	NA	NA	NA	NA	NA	NA	8514·64	8697·73	300·53	1·83
	1 tab	NA	285·50	NA	274·21	404·60	220·25	338·68	307·24	283·82	289·92		
Gefitinib 250 mg	30 film-coated tablets	NA	NA	NA	NA	NA	NA	NA	NA	3938.70	NA	144·33	2·40
	1 film-coated tab	NA	NA	173·85	123·84	157·52	82·79	198·89	142·14	131·29	NA		
Gemcitabine 1 g	Vial	295·81	210·77	496·53	227·33	179·04	271·28	359·10	256·57	281·60	NA	286·45	2·77
ImatinibMesylate 100 mg	60 film-coated tab	2485·80	5156·95	NA	NA	NA	NA	NA	NA	2920·71	3571·02	56·92	3·41
	1 film-coated tab	41·43	85·95	63.49	25·23	74·14	41·43	72·47	NA	48·68	59·50		
Interferon Alpha - 2B 1 m IU/injection	1 multidose pen	208·81	NA	NA	21·02	NA	26·80	NA	72·71	65·69	287·50	113·76	13·68
Lapatinibditosylate 250 mg	70 film-coated tablets	3341.73	1926·91	NA	NA	NA	NA	NA	NA	2526·36	4190·34	40·08	2·68
	1 film coated tab	47.74	27·53	NA	32·39	22·30	NA	54·68	NA	36·09	59·86		
Nilotinib 150 mg	112 caps	NA	2152·47	NA	NA	NA	NA	NA	NA	7906·28	9474·43	66·07	5·56
	1 cap	NA	19·22	NA	59·16	70·76	43·68	106·95	73·62	70·59	84·59		
Nilotinib 200 mg	112 caps	15,113·64	2982·06	NA	NA	NA	NA	NA	NA	11,069·25	12,631·82	92·78	5·07
	1 cap	134·94	26·62	116·07	NA	NA	67·44	NA	NA	98·83	112·78		
Paclitaxel Albumin 5 mg/ml	1 vial	213·07	NA	125	624·90	242·70	91·86	348·21	NA	NA	NA	274·29	6·80
Panitumumab 20 mg/ml	1 vial	NA	4932·74	NA	NA	NA	NA	7223·84	1291·00	5804·39	1482·96	4147·00	5·60
Pazopanib 200 mg	30 tab	NA	762·33	NA	NA	NA	NA	NA	NA	1604.18	1994·89	52·20	3·19
	1 tab	NA	25·411	NA	36.92	51·59	44·80	81	57·90	53·47	66·496		
Pemetrexed Disodium Heptahydrate 500 mg	1 vial	2485·80	3295·96	694·44	624·77	795·70	502·78	NA	2879·24	3200·21	3307·96	1976·32	6·58
Sorafenib 200 mg	6 × 10’s tablets	8267·05	5025·56	NA	NA	Na	NA	NA	NA	4920·29	6620·46	97·23	1·91
	1 tab	137·78	83·76	NA	NA	NA	72·27	NA	NA	82·00	110·34		
Sunitinib malate 12.5 mg	30 caps	3597·66	2578·48	NA	NA	NA	NA	NA	NA	3323·22	3425·57	108·22	1·75
	1 cap	119·92	85·95	150·47	NA	89·2	87·03	NA	NA	110·77	114·19		
Sunitinib malate 25 mg	30 caps	7121·80	7134·08	NA	NA	NA	NA	NA	NA	6646·44	6851·71	231·28	1·07
	1 cap	237·39	237·80	NA	NA	NA	NA	NA	NA	221·55	228·39		
Sunitinib malate 50 mg	30 caps	13,943·18	10,762·33	NA	NA	NA	NA	NA	NA	13,292·89	13,703·98	405·56	1·49
	1 cap	464·77	358·74	NA	NA	NA	312·31	NA	397·62	443·096	456·80		
Trastuzumab 440 mg	1 vial	4616·48	4686·10	NA	NA	NA	3930·91	NA	NA	NA	NA	4779·35	1·50
Transtuzumab 150	1 vial	NA	NA	NA	NA	1240·85	NA	2599·53	1857·33	5883·89	2133·52	1957·81	2·09
Zolendronic acid 4 mg/5 ml	1 vial	674·72	605·38	NA	NA	268·79	NA	NA	NA	NA	845·45	598·59	3·15
Mean unit price for every country		708·75	919·39	1803·51	990·30	658·09	492·61	2355·60	789·26	1496·34	607·13		
Average unit price by world bank categories		814.07		1150.63			1148.19						

### Data sources

The price of the cancer drugs in the ten included countries was retrieved from official pricing authorities or the respective Ministry of Health or equivalent websites [Table 1]. The authors ensured that the prices were retrieved from the most recent price lists published by the respective countries. The prices retrieved for Bahrain, Lebanon, Oman and Taiwan were published in 2016 and the prices for the remaining countries were published in 2015. However, the exact publication date of the pricing data was unclear.

The GNI per capita [USD] of nine of the ten included countries was retrieved from the World Bank website. For Taiwan, however, the data had to be retrieved from Taiwan’s national statistics bureau [10] since the country is not a member of United Nations and was not listed on the World Bank website. The respective GNI per capita and country classifications are shown in Additional file 1: Table S2.

### Medicine selection

A cross–country comparison of 31 selected samples of cancer drugs was made by Vogler et al. [7] whereby the selection of drugs was also dependent on the data availability and availability of comparable products in the market in at least ten countries. In this study, the same 31 drug presentations were chosen initially as a guide. The inclusion criterion for selecting a drug presentation was the availability its pricing data in at least four out of the ten countries.

### Data analysis

The price data were reviewed by the six co-authors. As the unit of measurement, we selected retail price per unit [i.e. per tablet/capsule or per vial]. Retail prices were chosen in this study because they represent the patients’ actual out of pocket expenditure. Another advantage of using retail prices is that they include all the add-ons such as taxes, distribution and pharmacist fees; these add-ons sometimes lead to the final price costing more than double the actual cost of the drug [11, 12]. The other reason for the use of retail prices was that ex-factory prices are hard to measure accurately, especially in countries that have no publicly funded drug coverage. [12]

Prices were only included if they referred to the same presentation in terms of pack size, strength and pharmaceutical form. Pricing data were originally extracted and presented in national currencies but later converted to USD using purchasing power parity (PPP) to allow fair comparison between different countries. Microsoft Excel was used for PPP-related calculations. Purchasing power parity rates were retrieved from the World Bank website [8]. The purchasing power parity conversion rates used are presented in Additional file 1: Table S1. The mean unit prices were calculated for each drug presentation as well as for each country. The high/low ratio for each presentation was calculated by dividing the highest unit price of the presentation by the lowest unit price of the same presentation to analyze the inter-country variability of the price for every presentation. A high/low ratio of 10 implies that the highest unit price is ten times more expensive than the lowest unit price. All calculations were performed in Microsoft Excel. All statistical analyses were descriptive.

In order to present the price comparison more effectively, the results have been arranged into the World Bank categories and comparisons are made accordingly. Of the ten included countries, one country [Pakistan] is classified as a low-income country, one [Egypt] as middle-income, three [Thailand, Malaysia and Lebanon] as higher-middle income, and five [the United Arab Emirates, Bahrain, Saudi Arabia, Oman and Taiwan] as high-income countries.

## Results

Upon applying the selection criteria for presentations, the following presentations that were originally in Vogler et al.’s study [7] were excluded from this study due to a lack of pricing information for at least four countries: bendamustine [HCl], bevacizumab [16 ml vial], clofarabine, eribulin mesylate, lenalinomide, nelarabine, ofatumumab, plerixafor, sorafenib [112-tablet pack], temsirolimus and vemurafenib. On the other hand, five additional presentations for which price data was available in the included regions were added to the list: nilotinib 200 mg, sunitinib 12·5 and 25 mg, trastuzumab 440 mg, and sorafenib [60-tablet pack].

We were unable to find pricing data on sorafenib [the 112-tablet box] so we replaced it with the 60-tablet box for which pricing data was available. Moreover, we found adequate data for trastuzumab 440 mg in the regions of interest, therefore we added it to the list despite already having a presentation for this drug [trastuzumab 150 mg]. Although it was expected that the two would follow the same pattern, the pricing data was included to provide more information for the readers. The same was true for sunitinib [12·5 mg, 25 mg and 50 mg] and nilotinib [150 mg and 200 mg].

The final number of included presentations was 26. Table 2 provides an overview of the selected drugs with regards to their FDA-approved indications, selected presentation and country coverage. We only compared prices for the originator drugs because pricing policies for originator drugs differ substantially from generic vials.

### Unit price

Table 3 represents prices per package as well as the calculated unit prices in USD using PPP. Upon estimating the PPP, cabazitaxel was the most expensive drug with a unit price of $17,304·9/vial [in Oman]. Nilotinib 150 mg had the lowest unit price in USD [$19·22/tab in Egypt].

**Fig. 1:**
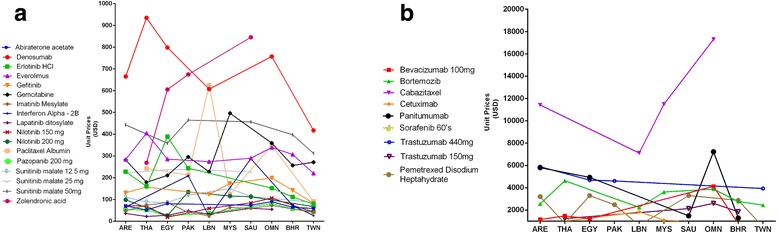
**a** Included cancer drugs with unit prices between 1-1000 USD stratified by country. **b** Included cancer drugs with unit prices between 1000-20,000 USD stratified by country

The PPP adjusted USD unit prices for each medication are represented in Fig. 1. The different unit prices are connected to illustrate variations between countries. To ensure that the variations among prices is easy to discern for both low price and high price medications, the two have been presented in two different figures. Figure 1a represents cancer drugs with unit price between $0–1000, while Fig. 1b represents cancer drugs with unit prices between $1000–20,000.

### Mean unit price

Six presentations [23·1%] had a mean unit price below $100.00 and nine drugs [34·6%] had a mean unit price between $100·00 and $500·00. Eight drugs [30·7%] had a mean unit price of over $1000.00, of which, one [cabazitaxel] had a mean unit price of over $5000·00 [$11,832·9/vial]. Overall, Taiwan had the lowest mean unit prices[$492·61] and Oman the highest [$2355·6]. So Overall, Taiwan had the lowest mean unit prices for all presentations.

The average unit prices by country income category were as follows: LMICs $814.07, HMICs $1150·63, HICs: $1148·19. Using PPP-adjusted mean unit prices, the three most expensive presentations were found to be cabazitaxel [$11,832·93], trastuzumab 440 mg [$4779·35], and panitumumab [$4146·99]. The three cheapest oncology drugs were lapatinib ditosylate [$40·08], pazopanib disodium heptahydrate [$52·20], and imatinib [$56·92].

### The high/low ratio

The high/ low ratio data included in Table 3 allowed us to look at the price deviation between countries. The high/low unit price ratio was less than 3 for fourteen drugs [53·80% of the 26 total included products], between 3 and 6 for eight drugs [30·77%] and more than 6 for four drugs [15·38%]. The smallest high/low ratio was 1·07, which belonged to sunitinib malate 25 mg, and the largest high/low ratio belonged to Interferon alpha-2B [13·68] [Table 3].

### The frequency of unit prices ranked in quartiles

**Fig. 2:**
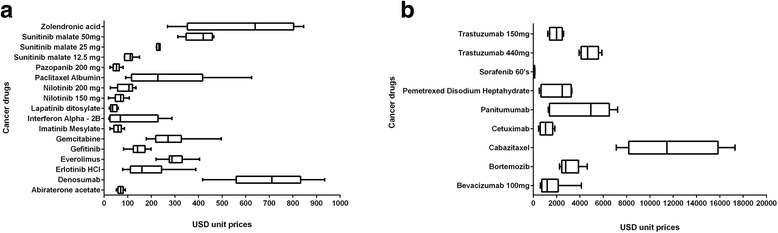
**a** Boxplots of incuded cancer drugs with mean USD unit price between 1 and 1000. **b** Boxplots of included cancer drugs with mean USD unit price between 1,000 and 20,0000

Box plots have been constructed based on the unit $ price of the included drugs. The boxplot for low and high price cancer medicines have been presented separately in Fig. 2a and b to ensure that the differences are easy to discern. The box plot displays the inter-quartile range [IQR] as calculated by Microsoft Excel; the bottom and top of the box are the 25th and 75th percentiles [the 1st and the 3rd quartiles, respectively], and the band in the middle of the box is the median. The extended lines describe the bottom and top whiskers.

**Fig. 3:**
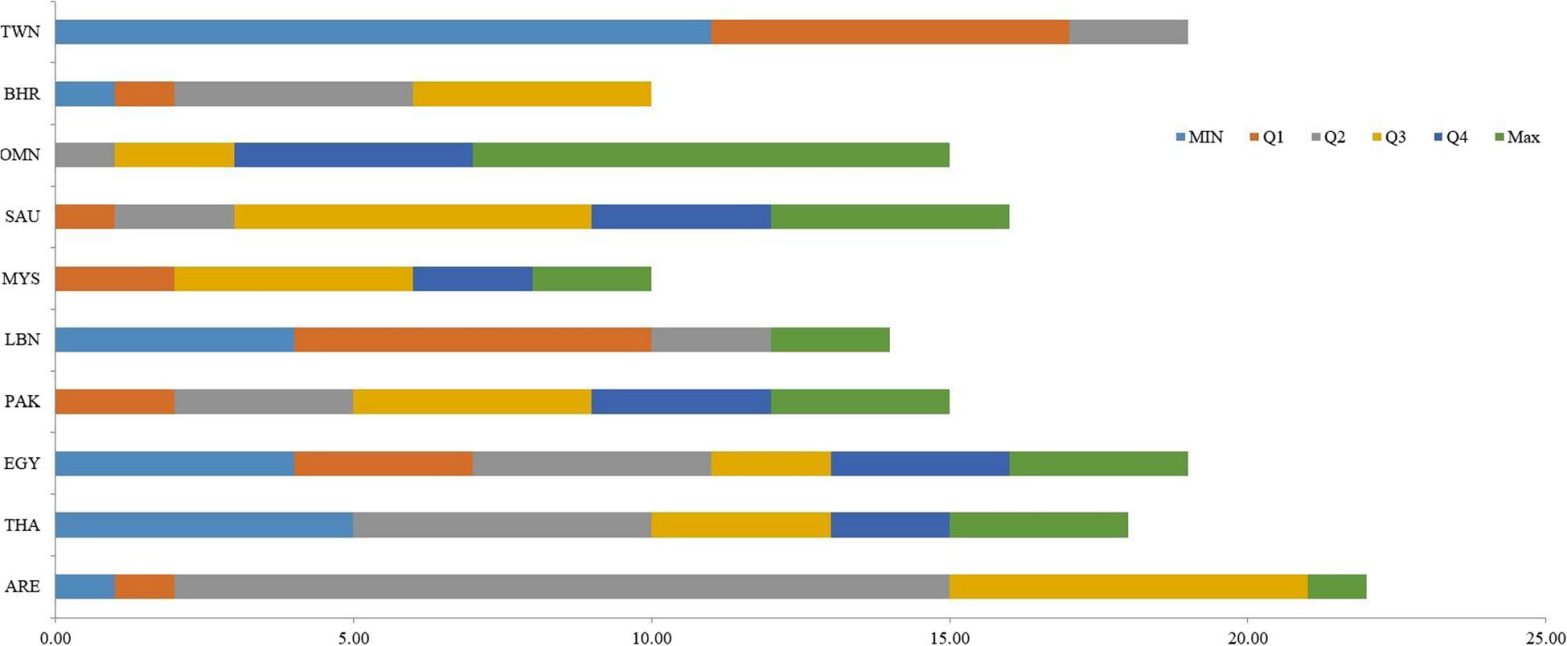
Frequency of PPP-adusted unit prices ranked in quartiles for each country

Drug prices varied significantly across the included countries. Figure 3 represents the frequency of unit prices, as of February 2016, ranked in quartiles [Note: quartile 1 and 4 are not inclusive of the minimum and maximum values; these values have been categorized and represented separately using unique colours of their own]. Thailand and Taiwan had prices at the lower end, prices in Lebanon mainly fell in the first quartile, while prices in the United Arab Emirates and Bahrain fell in the second and third quartiles. Prices in Oman and Saudi Arabia were at the upper end [Fig. 3]. Prices in Oman were ranked the most expensive for eight presentations (Additional file 1: Table S3).

**Fig. 4:**
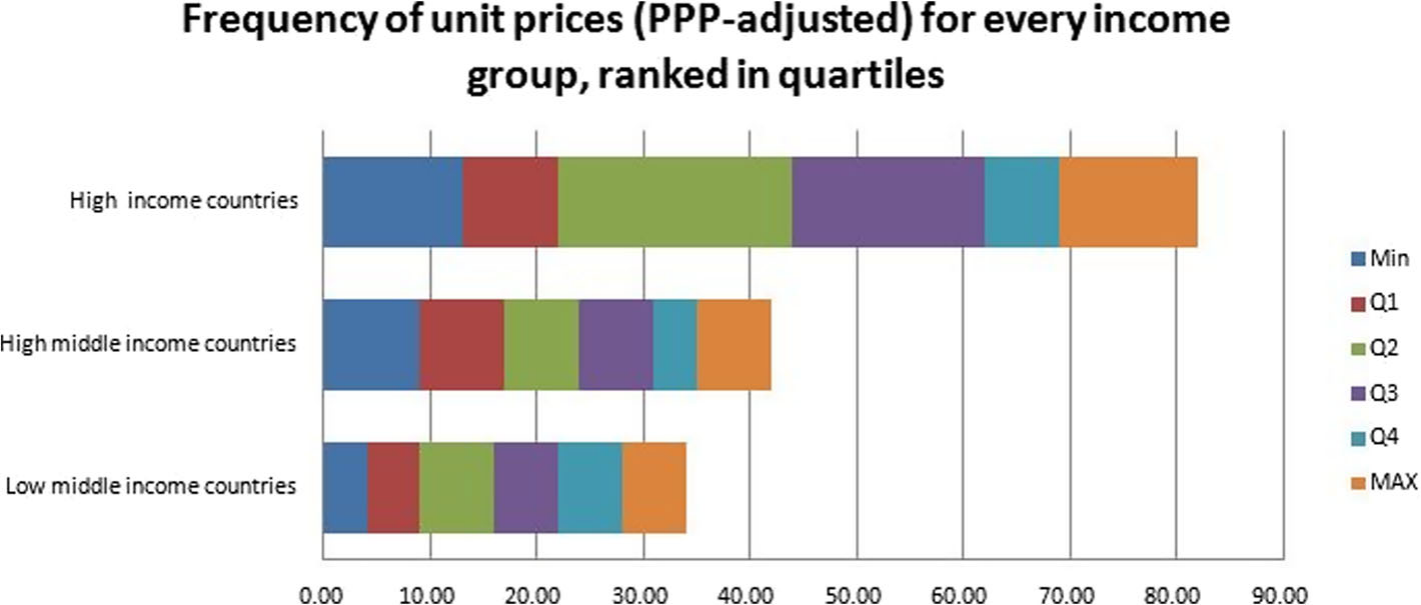
Frequency of PPP-adjusted unit prices ranked in quartiles for each income group

Prices in high income countries are at the upper end, and are ranked as the maximum for 13 of the included presentations, while prices in low middle income countries were ranked the most expensive for six presentations only [Fig. 4].

## Discussion

The aim of the present study was to survey and compare retail prices of anti-cancer drugs between high, middle and low-income countries in the South-East Asia, Western Pacific and Eastern Mediterranean regions. In the absence of a systematic pricing system, pharmaceutical companies determine the drug price according to what the society can afford, as people are ready to face a heavy financial burden when it comes to treating fatal diseases [13]. In some developed countries, price regulation measures such as external reference pricing or international reference pricing have been widely used by policymakers to restrain drug costs. External reference pricing is defined by the WHO as: “*The practice of using the price[s] of a medicine in one or several countries in order to derive a benchmark or reference price for the purposes of setting or negotiating the price of the product in a given country”* [14].

A list of 2010 cancer drug prices, has been published by the Management Sciences for Health based on the WHO’s 17th edition of the Essential Medicines List [15]. This is the only procurement tool available to the pricing authorities in LMICs, however, more support is needed such as an updated WHO essential medicine list section on oncology drugs along with cross-country pricing information and procurement guidance. Although the Western Pacific Region office of the WHO has developed a Price Information Exchange that provides comparative information on procurement prices for selected medicines across the Western Pacific region [15], it has faced many challenges such as lack of cooperation from member countries. This is why we have attempted to undertake our own cross-country price comparison [13].

Purchasing power parity conversion rates were used instead of exchanges rate in this review. Exchange rates determine the producers’ actual profit from foreign sales in terms of domestic currencies [16], and can be used when performing pharmaco-economic studies within a country since the expected exchange rate fluctuations would affect all drugs sourced from the same country, uniformly. In cross-country comparisons, however, to overcome the effect of large fluctuations in exchange rates, the PPP is used as an alternative sensitivity analysis [17]. Purchasing power parity conversions are also argued to be more apt for comparisons at final consumer level [16].

Our review showed extreme variation between drug prices across countries; the high/low ratios were as high as 13·68 [PPP-adjusted]. While patents can explain the price differences between drugs, they are not responsible for the price differences observed for the same medicine.

Greater transparency of price information among countries may assist with in-country negotiations between purchasers and suppliers. Information on the availability of cheaper medicines in neighbouring countries has the potential to encourage policy and managerial decisions at national levels in an effort to reduce prices [15]. Economic evidence on the impact of external reference pricing is scarce. Only a few studies have explicitly analysed the impact of external price referencing on medicine prices. Stargardt et al., [18] using an analytic model to simulate the effect of a price reduction in Germany demonstrated that every 1 EURO reduction in price in Germany would lead to a reduction of EURO 0.15 to EURO 0.36 in 15 European countries that use external reference pricing. Similar results were reported by Windmeijer et al. [19] who investigated the result of external reference pricing implementation on prices in Netherlands. Our study can hence be used by officials to improve access to cancer treatment [13].

### Limitations

Cross-country comparison of pharmaceutical prices is challenging because of the differing level of sales, frequent changes in exchange rates and the differences in the pharmaceutical presentations such as strength, pack size, dose and dosage form. Of the 57 countries in the South-East Asia, Eastern Mediterranean and Western Pacific regions (excluding Australia and New Zealand), we only managed to find reliable pricing information for ten countries only due to: [i] under-developed/incomplete/not-user-friendly websites of official pricing/heath authorities; [ii] use of languages other than English in official websites; [iii] lack of public access to official drug prices; and [iv] absence of an official institution to summarize/compare prices across Asia. However, we managed to include representative countries from different ranges of GNI per capita. The other key limitations of this study are as follows: Firstly, the prices may not reflect the true cost of medications because the retrieved data are the official prices as published by the pricing authorities without consideration of [usually confidential] discounts and rebates. Secondly, this study used retail prices, which include add-ons such as taxes and distribution fees. Understanding of the amount and sources of add-ons would identify potential targets for price reduction. Unfortunately, data on add-ons was limited and hence we were unable to estimate them. Thirdly, the use of PPP calculations for price comparison required the assumptions that the value of goods and services are homogeneous across countries and that international shipment of goods takes place instantaneously, and with no cost. Unit prices were used to compare results in this study, when interpreting the results of this study, it should be kept in mind that one unit may refer to the daily dose of a tablet, or monthly vial for injection or a weight based two weekly injections. Future studies should use the data provided in this study and perform a price comparison using monthly dose or total treatment cost, as a unit for measurement and comparison. Finally, pricing revisions are done at irregular intervals and the price lists may not be updated immediately. However, most recently available prices were used for calculations.

## Conclusion

The significant price differences among Asian countries is very evident. Taiwan had the lowest mean unit price [$492·61] and Oman the highest [$2355·60]. Significant variation between drug prices across countries with the highest high/low ratio was seen for Interferon alpha-2B: 13.68. Cabazitaxel was ranked the most expensive drug in our sample with a mean unit price of $17,304·95. These discrepancies indicate that greater price transparency is required. Our goal was to compare cancer drug prices and investigate whether the prices are significantly different among countries. Significant differences were found and reported accordingly, however, what this price differences mean in terms of access to cancer medications, government spending, and patient adherence, requires a much more in-depth analysis of each country’s respective health care system, which was beyond the scope of this paper.

Our results can be used to help policy makers to compare the price of anti-cancer agents in their country with that in neighbouring countries to decide if further policy measures related to drug prices are required.

## Key issues

- Anti-cancer drug prices are highly variable in the South-East Asian, Western Pacific and Eastern Mediterranean regions.
- There is an association between price of anticancer drugs and income category of the country.
- Almost one in three drugs assessed in this study had a mean unit price of more than US$ 1000.
- There is a need to review pricing policy in order to improve accessibility and affordability of cancer drugs in the selected countries.

## Additional file

Additional file 1: Table S1. The purchasing power parity conversion rates used. **Table S2.** The respective GNI per capita and country classifications. **Table S3.** (XLSX 72 kb)
